# *Toxoplasma gondii* in Slaughtered Sheep in High- and Low-Humidity Regions in the South of Iran: Molecular Prevalence and Genotype Identification

**DOI:** 10.1155/2021/5576771

**Published:** 2021-07-05

**Authors:** Seyedeh Zahra Khademi, Fatemeh Ghaffarifar, Abdolhossein Dalimi, Mohammad Saaid Dayer, Amir Abdoli

**Affiliations:** ^1^Department of Biology, Payam Noor University (PNU), Tehran, Iran; ^2^Department of Parasitology, Faculty of Medical Sciences, Tarbiat Modares University, Tehran, Iran; ^3^Zoonoses Research Center, Jahrom University of Medical Sciences, Jahrom, Iran; ^4^Department of Parasitology and Mycology, School of Medicine, Jahrom University of Medical Sciences, Jahrom, Iran

## Abstract

*Toxoplasma gondii* is one of the most common meat-born zoonoses that infect all warm-blooded animals and humans. Sheep (*Ovis aries*) is one of the main reservoirs of *T. gondii* worldwide, and the infections induce various sequels, such as abortion and stillbirth. The present study aimed to identify the effects of humidity on the prevalence of *T. gondii* in sheep in high- and low-humidity regions. Heart samples from 200 slaughtered sheep (140 samples from a high-humidity region and 60 samples from a low-humidity region) were collected from Hormozgan Province (south of Iran). The samples were tested by nested PCR targeting the RE gene. Genotyping was performed by the PCR-RFLP method using the SAG3 and GRA6 genes. Some isolates were sequenced and recorded in the GenBank. *T. gondii* DNA was detected in 10.71 percent of the samples from the highly humid region, whereas no positive samples were detected in the low-humidity region. Genotyping revealed that all isolates belonged to the *T. gondii* type III genotype. Our study indicated that humidity is an important factor for the prevalence of *T. gondii* in sheep. Additionally, our study also showed the dominance of type III strain of *T. gondii* in sheep in the south of Iran.

## 1. Introduction

*Toxoplasma gondii* is one of the most common meat-born parasitic infections worldwide. Domestic cats are the final host, and all warm-blooded animals and humans are intermediate hosts of *T. gondii*. The infection can be occurred by consumption of raw or undercooked meat containing tissue cysts, drinking or eating contaminated water or foods with oocysts (shed in by cat feces), and maternal transmission or rarely blood transfusion or organ transplantation [1–3]. Typically, the infection is asymptomatic in healthy individuals; however, severe infection can be developed in immunocompromised patients (e.g., HIV/AIDS, cancer, and organ recipient patients) [2, 4, 5]. Congenital infection is one of the major sequels of the toxoplasmosis in human and animals that may lead to several sequels for the mother and fetus, such as pregnancy complications, stillbirth, fetal loss, abortion, and neuropsychiatric symptoms in the offspring [1, 6–8].

Environmental conditions, people's social behaviors, and fauna are some of the factors that may determine the level of contamination with *Toxoplasma gondii*, so the parasite is more prevalent in areas with hot and humid climate than in dry and cold areas [9]. The main routes of transmission of *T. gondii* are via consumption of raw or semicooked meat of animals such as sheep, pigs, and cattle infected with tissue cysts [3]. Sheep (*Ovis aries*) is an important reservoir of *T. gondii* worldwide. Toxoplasmosis in sheep induces various sequels, such as abortion and stillbirth, and, therefore, has economic and public health importance [10, 11].

The pathogenicity of *T. gondii* depends on various factors including the host immune system, genetic background, and the type of parasite. One of the important factors which affect the virulence of toxoplasmosis is the parasitic type [12, 13]. *T. gondii* has three main types; type I, II, and III, and each of them has different virulence and induces different degrees of disease severity [14]. Type I has the highest virulence and causes severe infections in humans. Type II is common in humans and animals and most often causes chronic illness, although it can be severe in patients with poor immune systems. This type has been isolated from patients with congenital and ocular toxoplasmosis [14, 15]. Type III of *T. gondii* is less virulent than the other two but highly prevalent in birds [15].

A recent study among pregnant women in Hormozgan Province of Iran revealed a significant relationship between *T. gondii* seroprevalence and consumption of raw or half-cooked meat and history of contact with cats [8]. The present study aimed to determine the prevalence of toxoplasmosis in sheep in two warm regions (humid and dry) of Hormozgan Province (south of Iran) and to determine the common genotypes of *T. gondii* by the PCR-RLFP technique.

## 2. Materials and Methods

### 2.1. Sample Collection

The heart samples from slaughtered sheep were collected from 2 different areas, Bandar Abbas and Hajiabad (Hormozgan Province, south of Iran). The former region is located at coordinates of 27°15'N-56°15'E at 9.4 m above sea level and has a hot and humid climate with seasonal temperatures ranging from 2 to 51°C and the average relative humidity of 64.8 per cent. The latter is located at coordinates of 28°19'N-55°55'E at 1200 m above sea level with seasonal temperatures ranging from −5 to 40°C and has an average humidity of 25 percent. Since few molecular studies have been performed on the prevalence of toxoplasmosis in Iranian sheep, in this study, we used the global prevalence of parasites in sheep tissues (14.7%) to determine the sample size [16]. In this study, heart samples were collected from 200 slaughtered sheep (140 samples from the Bandar Abbas slaughter house and 60 samples from local butcheries in Hajiabad). Then, we selected 200 samples, and according to the sheep population in the two areas, the 200 samples were divided to 140 samples for Bandar Abbas and 60 samples for Hajiabad.

### 2.2. DNA Extraction from Sheep Heart Samples

Approximately 100 mg of each heart sample was collected and placed into 1.5 ml microtubes, and DNA was extracted by using the Sina Clone Extraction Kit (Cat No. PR881613).

### 2.3. PCR of the Repetitive Element (RE) (529 bp) Gene

PCR was carried out using primers (Table 1) of the 529 bp Repetitive Element (RE) gene in a total volume of 20 *μ*l of solution containing master mix 10 *μ*L (Ampliqon, Denmark, Cat. No. A170301), MgCl_2_, 2 mM, primers 2 *μ*L (pmol 10), DNA 2 *μ*L, and water [18]. The PCR reaction was carried out with an initial denaturation temperature of 94°C (30 sec), 35 cycles of 94°C (30 sec), annealing of 56°C (30 sec), and extension of 72°C (30 sec) followed by the final extension at 72°C (10 min). The PCR products were resolved on 1.5 percent agarose gel in parallel with a 100 bp ladder. Nested PCR for the GRA6 gene: Following the primary PCR results, all RE-positive heart samples were reamplified by the GRA6 gene using two pairs of nested PCR primers (Table 2). The first PCR reaction consisted of 10 *μ*L of Master Mix X2 Amplicone (Ampliqon, Denmark, Cat. No. A170301) with a concentration of 2 mM MgCl_2_, 2 *μ*L of primer (10 pmol), and about 1.5 to 2 *μ*L of DNA with the addition of sterile injectable reaction water up to a final volume of 20 *μ*L. Nested PCR was also performed in the final volume of 20 *μ*L containing the substances mentioned in the first step, with the addition of 1 to 1.5 *μ*l of the first PCR products. In the first PCR and nested PCR, a positive control sample and a negative control sample were also included. The PCR and nested PCR products were resolved on 1.5 percent agarose gel in parallel with a 100 bp ladder using TAE buffer.

### 2.4. Nested PCR for SAG3 and GRA6 Genes

All RE-positive heart samples were analysed by the SAG3 and GRA6 genes using their specific primers (Table 2). The first PCR reaction consisted of Master Mix X2 amplicon 10 *μ*L (Denmark, Cat. No. A170301 Ampliqon), MgCl_2_ 2 mM, primer (10 pmol), 2 *μ*L and 1.5 or 2 *μ*L of DNA, and sterile injectable water up to the final volume of 20 *μ*L. Nested PCR was also performed in the final volume of 20 *μ*L containing the substances mentioned in the first step, except that 1 *μ*L of the first PCR product was added to the reaction instead of DNA and the final volume (20 *μ*L) was made using sterile distilled water. In addition to each reaction in the first PCR and nested PCR, a positive control sample and a negative control sample were also used. PCR and Nested PCR products were resolved on 1.5 percent agarose gel in parallel with a 100 bp ladder using Tris-acetate-EDTA (TAE) buffer.

### 2.5. RFLP by the GRA6 Gene

The positive isolates obtained by the GRA6 gene were digested by the RFLP method using MseI (Tru1I) restriction enzyme according to Thermo Fisher Scientific Cat. No. ER 0982. The reaction at the final volume of 16 *μ*L consisted of 5 *μ*L of PCR2 product and 1 *μ*L of enzyme, 1 *μ*L of buffer (X10) R, and 9 *μ*L of injectable distilled water. To prevent evaporation, the material was completely covered with parafilm, and then, the tubes were placed in a bain marie at 65°C for 4 hours. The PCR product was then resolved on 3 percent agarose gel in TAE buffer [18, 19]. To obtain the RFLP pattern of the GRA6 gene, sequences of three known types of RH type I, ME49 type II, and NED type III parasites were obtained from GenBank and enzyme online at nebcutter (http://nc2.neb.com/NEBcutter2) [19].

### 2.6. RFLP by the SAG3 Gene

The positive isolates obtained by the SAG3 gene were digested by the RFLP method using NciI (BcnI) restriction enzyme according to the enzyme procedure (Thermo Fisher Scientific, USA, Cat. No. ER0061). The reaction at the final volume of 16 *μ*L consisted of 5 *μ*L of PCR2 product and 1 *μ*L of enzyme, 1 *μ*L of tango buffer (X 10), and 9 *μ*L of injectable distilled water. To prevent evaporation, the material was completely covered with parafilm, and then, the tubes were placed in a bain marie at 37°C for 10 hours. The PCR product was then resolved on 3 percent agarose gel in TAE buffer [18, 19].

### 2.7. Sequencing

Nested PCR products of the SAG3 and GRA6 genes were amplified in high volumes using primers of both genes and forwarded to Macrogen, South Korea, via Pioneer Company for sequencing. The sequences were then modified by Sequencher software and aligned with BioEdit Multiple Alignment software. The obtained sequences were recorded in the GenBank and compared with other recorded sequences.

## 3. Results

### 3.1. *T. gondii* Detection by PCR

**Figure 1:**
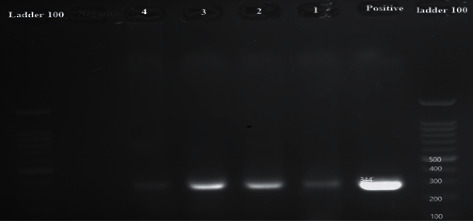
Gel electrophoresis of positive and negative samples and samples of hearts by the 344 bp GAR6 gene: from the right, 100 bp marker, positive control sample, respectively, lines 1 and 2 positive heart samples, and negative and positive control ladder 100 bp.

**Figure 2:**
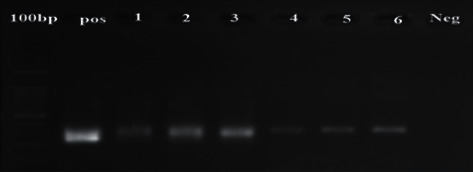
Gel electrophoresis of positive and negative sheep heart samples by the SAG3 gene: left marker 100 bp, positive control sample, positive sheep heart (lanes 1–6), and negative control.

Out of 200 heart samples collected and tested by the 529 bp RE gene, 15 samples (7.5%) were positive for toxoplasmosis. They all were collected from Bandar Abbas and represented 10.71 percent of the corresponding samples, whereas none of those obtained from Hajiabad were positive. The nested PCR performed on the RE-gene-positive samples using GRA6 and SAG3 genes resulted in the production of positive fragments of 344 bp (Figure 1) and of 224 bp (Figure 2), respectively.

### 3.2. Genotyping of *T. gondii*

**Figure 3:**
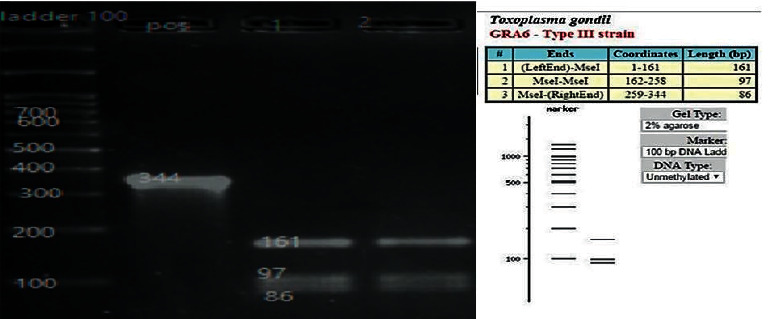
Gel electrophoresis of the *T. gondii*-isolated GRA6 gene by Tru1I enzyme: left marker 100 bp, positive sample, and lines 1 and 2 sheep heart that showed type III pattern.

**Figure 4:**
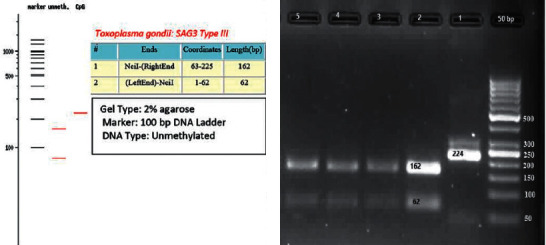
Gel electrophoresis of the *T. gondii*-isolated SAG3 gene by NciI enzyme: 1, positive sample; 2–4, sheep heart samples; and 5, positive control that showed type III pattern.

The amplified fragments of the GRA6 gene were 73.3 percent (11 of 15), and those of SAG3 gene fragments were 67 percent (8 of 15). RFLP analysis of the GRA6 gene with MseI (Tru1I) restriction enzyme revealed three fragments of size 161, 97, and 86 bp indicating type III genotype (Figure 3). All the isolates belonged to the type III genotype. RFLP analysis of the SAG3 gene with NciI (BcnI) restriction enzyme revealed two fragments of size 162 and 62 bp (Figure 4) indicating the type III genotype. All the isolates belonged to the type III genotype.

### 3.3. Sequencing

Two isolates were sequenced by the SAG3 gene and deposited in the GenBank database (MF939105 and MF939106). The nucleotide blast of these sequences at the NCBI (http://blast.ncbi.nlm.nih.gov/blast/Blast.cgi) revealed 100 percent similarity with *T. gondii* isolated that was deposited in the GenBank.

## 4. Discussion

Humans are mainly infected by toxoplasmosis via eating raw or undercooked meat containing parasitic tissue cysts [21]. The most susceptible animals to infection are sheep, goats, and pigs that play an important role in transmitting the parasite to humans [21]. The serological prevalence of *T. gondii* in sheep has been reported to be about 30 per cent [10, 23], and active infection reported to be about 15 percent worldwide [16]. This is largely depending on the climate and the kind of livestock utilized for breeding. The results of a meta-analysis study of the prevalence of *T. gondii* by the molecular method or injection into mice showed that the prevalence of parasites in sheep tissues was 14.7 percent (8 : 21.5) (Cl) around the world [16]. Till now, no study has been carried out on toxoplasmosis in sheep bred under two climatic conditions in locations with approximately the same geographical coordinates but one having warm-high humid climate and the other having warm-low humid weather. The prevalence of toxoplasmosis in highly humid climate was 10.71 percent as revealed via molecular techniques, whereas no positive case was detected in samples taken from warm and less humid climate.

A few studies have been performed in Iran to detect *T. gondii* infection using molecular methods [24–26], but little has concentrated on the characterization of *T. gondii* genotypes in sheep. Rasti et al. [26] detected the *T. gondii*'s DNA in 17.8 percent of sheep and 8.9 per cent of goat heart samples in the center of Iran, Kashan. Our previous study on *T. gondii* infection in eggs carried out in the Hajiabad region showed no contamination. The facts indicate that humidity plays an important role in the spread of toxoplasmosis [18]. Genotyping of 57 isolates obtained from sheep in the USA resulted in 45.6 percent of type II, 15.7 per cent of type III, and 22 atypical isolates [27]. Genotyping of 22 isolates obtained from the sheep brain, heart, diaphragm, and lung in Brazil identified 13 isolates to be of type II and III, while the rest could not be characterized [28]. Genotyping of *T. gondii* isolates obtained from sheep, cattle, and pigs in Switzerland showed that type II genotypes were the most common parasite in sheep, whereas group III, I, and unusual genotypes were more common in cows and pigs [29]. In England, 21 samples of contaminated meats included parasites characterized to be of genotype I and six others contained both types I and II [30]. In the present study, all samples were identified to be type III isolates. Genotyping of positive cases using the PCR-RFLP method showed that all samples were infected with type III *T. gondii*.

Our main finding was the role of humidity in the epidemiology of *T. gondii* in sheep. Hence, finding risk factors of *T. gondii* in humid regions could help in the prevention of toxoplasmosis in sheep.

## Figures and Tables

**Table 1:** Primer sequences for the 529 bp RE gene.

Test	Primer	Sequence	Length	Reference
PCR RE	Forward	5'-CGCTGCAGGGAGGAAGACGAAAGTTG-3'	26	Homan et al. [17]
	Reverse	5'-CGCTGCAGACACAGTGCATCTGGATT-3'	26	

**Table 2:** Primer sequences for GRA6 and SAG3 genes for nested PCR.

Marker	Primer	External primers (5'–3')	Internal primers (5'–3')	Ref
GRA6	Forward	ATTTGTGTTTCCGAGCAGGT	TTTCCGAGCAGGTGACCT	Abdoli et al. [19]
	Reverse	GCACCTTCGCTTGTGGTT	CGCCGAAGAGTTGACATAG	

SAG3	Forward	CAACTCTCACCATTCCACCC	TCTTGTCGGGTGTTCACTCA	Su et al. [20]
	Reverse	GCGCGTTGTTAGACAAGACA	CACAAGGAGACCGAGAAGGA	

## Data Availability

No additional data were used to support this study.
